# Insulin‐Like Growth Factor Binding Protein 2 Predicts Right Ventricular Reverse Remodeling and Improvement of Concomitant Tricuspid Regurgitation After Transcatheter Edge‐to‐Edge Mitral Valve Repair

**DOI:** 10.1002/clc.70048

**Published:** 2024-11-26

**Authors:** Matthias Gröger, Dominik Felbel, Michael Paukovitsch, Leonhard Moritz Schneider, Sinisa Markovic, Wolfgang Rottbauer, Mirjam Keßler

**Affiliations:** ^1^ Department of Internal Medicine II Ulm University Heart Center Ulm Baden‐Württemberg Germany

**Keywords:** biomarker, mitral valve repair, prognosis, TEER, tricuspid regurgitation

## Abstract

**Background:**

Concomitant right ventricular (RV) failure and tricuspid regurgitation (TR) are common comorbidities in patients undergoing mitral valve transcatheter edge‐to‐edge repair (M‐TEER) and are associated with worse prognosis. Improvement of TR after M‐TEER occurs frequently, however determinants of this course are poorly understood. This study aimed to analyze serum biomarkers that are differentially regulated in patients with TR and to identify biomarkers predictive of the course of TR after M‐TEER.

**Methods and Results:**

Biomarker expression was analyzed in 242 prospectively included patients undergoing M‐TEER. Patients with moderate‐to‐severe TR had significant comorbidities (median EuroSCORE II 5.2 in patients with severe TR, 4.9 in moderate TR, 3.2 in no/mild TR; *p* = 0.002) and a large number of biomarkers was upregulated including IGFBP‐2 (1.4‐fold in severe TR compared to no/mild TR, *p* = 0.005). Echocardiographic follow‐up 3 months after M‐TEER was carried out in 99 patients. RV reverse remodeling (RVRR) as defined by improvement of concomitant TR by at least one grade and/or RV diameter downsizing of at least 10% compared to baseline was seen in 50 patients (50.5%). IGFBP‐2 (Odds Ratio 2.078) and presence of chronic pulmonary disease (Odds Ratio 15.341) proved independent predictors of non‐development of RVRR within 3 months after M‐TEER.

**Conclusions:**

In patients undergoing M‐TEER with concomitant moderate or severe TR, numerous cardiometabolic biomarkers including IGFBP‐2 are upregulated. Higher levels of IGFBP‐2 at baseline are independently associated with persistent TR and/or RV dilation after M‐TEER.

## 1 Introduction

Percutaneous transcatheter edge‐to‐edge mitral valve repair (M‐TEER) is an effective interventional treatment of moderate‐to‐severe mitral regurgitation (MR) in symptomatic patients with high surgical risk and favorable anatomy [1]. Right ventricular (RV) dysfunction and concomitant tricuspid regurgitation (TR) are frequent comorbidities in patients undergoing M‐TEER and are associated with worse prognosis [2, 3, 4]. However, putatively due to reduction in left atrial and subsequently in pulmonary artery pressures by successful M‐TEER, reverse RV remodeling with decrease in TR severity occurs in up to 50% of patients [4, 5]. However, factors determining this postinterventional course have been poorly studied. Recent work has suggested, that while atrial fibrillation and RV dilation are preventive of TR improvement after M‐TEER [6], female sex as well as higher MR and TR grades at baseline are positively associated with TR regression [7]. To date, data on the role of biomarkers in the context of concomitant TR in patients undergoing M‐TEER are sparse. We therefore aimed to identify biomarkers associated with TR severity and course of TR after M‐TEER.

## 2 Methods

We prospectively enrolled 242 patients who were planned to undergo M‐TEER at our center from April 2017 to December 2020. Pre‐procedural peripheral blood samples for biomarker analysis were obtained from all study patients. A commercial multiplex protein assay (Cardiovascular III, Olink, Uppsala, Sweden) was carried out for expression level quantification of 92 defined biomarkers using an arbitrary base 2 logarithmic scale of normalized protein expression (NPX). With this scale, an absolute difference of 1.0 equals a twofold increase in protein expression.

All patients included in the present study were symptomatic in terms of heart failure (New York Heart Association [NYHA] functional class ≥ II) despite guideline‐directed medical therapy. A complete echocardiographic workup was performed in all patients. Severity of MR was classified in 4° according to the latest EACVI/ESC recommendations for MR quantification [8]. Post‐procedural MR severity was assessed by 2D and 3D transesophageal echocardiography after final device placement and removal of the guide catheter. MR was semi‐quantitatively assessed by visual estimation of MR jet area and by (biplane) determination of the Vena contracta of the major MR jet. In addition to MR severity, mitral valve gradients and area by 3D technique were assessed before and after device deployment and after removal of the guide catheter. Device success was defined as TEER with reduction of MR by at least one grade and absence of major device or procedure related serious adverse events [9].

RV diameters were measured at the base of the RV in the apical four‐chamber view in end‐diastole. Severity of TR at baseline and follow‐up was assessed by biplane vena contracta and/or effective regurgitation orifice area (EROA) by proximal isovelocity surface area (PISA) method and was classified in three grades [8]. Main study endpoint was RV reverse remodeling (RVRR) 3 months after M‐TEER. For this endpoint, only patients with at least moderate TR at baseline were included. Follow‐up visits were carried out at our center. RVRR was defined as TR reduction by at least one grade and/or decrease in basal RV diameter by at least 10% compared to baseline.

Left‐ventricular (LV) ejection fraction (LV‐EF) was measured using the biplane Simpson's method. LV diameters were measured in the parasternal long axis view. Systolic pulmonary artery pressure was determined invasively or—if no right heart characterization was performed—was estimated echocardiographically through TR velocity.

Statistical analysis was performed using SPSS 29 software (IBM Corp., Armonk, USA). Categorical variables are expressed as counts and percentages and were compared by Chi‐square test or McNemar test for longitudinal comparisons. Continuous parameters were analyzed for normal distribution using Kolmogorov‐Smirnov‐test. Normally distributed variables are presented as the mean ± standard deviation and were compared with *t*‐test. Variables without normal distribution are shown as the median and quartiles and were compared with Mann−Whitney‐*U*‐test. Kruskal−Wallis‐test was performed when comparing more than two continuous variables.

To determine predictors of RVRR, univariate logistic regression analysis was performed for all potentially influential variables (*p* < 0.2). In multivariate regression analysis, a backward stepwise algorithm was applied to all potentially influential parameters from univariate analysis (*p* < 0.2). Correlation was tested using Pearson's correlation coefficient. Variables with significant correlation (e.g., NT‐proBNP and insulin‐like growth factor binding protein 2 [IGFBP‐2]) were excluded from multivariate analysis.

All tests were two‐tailed and differences were considered statistically significant when *p* < 0.05.

The study was ethically approved by the ethics committee of the University of Ulm (Approval Number 435/16) and complied with the principles outlined in the Declaration of Helsinki (Br Med J 1964; ii: 177).

## 3 Results

### 3.1 Biomarkers in MR Patients With Concomitant TR

242 patients were analyzed before M‐TEER. 94 patients (38.8%) had mild concomitant TR or less (grade < I−I), 85 had moderate TR (grade II; 35.1%) and 63 had severe TR (grade III; 26.0%). Patients with more severe TR were older and had significant comorbidities resulting in higher EuroSCORE II and STS‐Score. Patients with higher TR grades also had more severe RV dilation. Baseline clinical characteristics of the three subgroups are shown in Supporting Information S1: Table 1.

**Figure 1:**
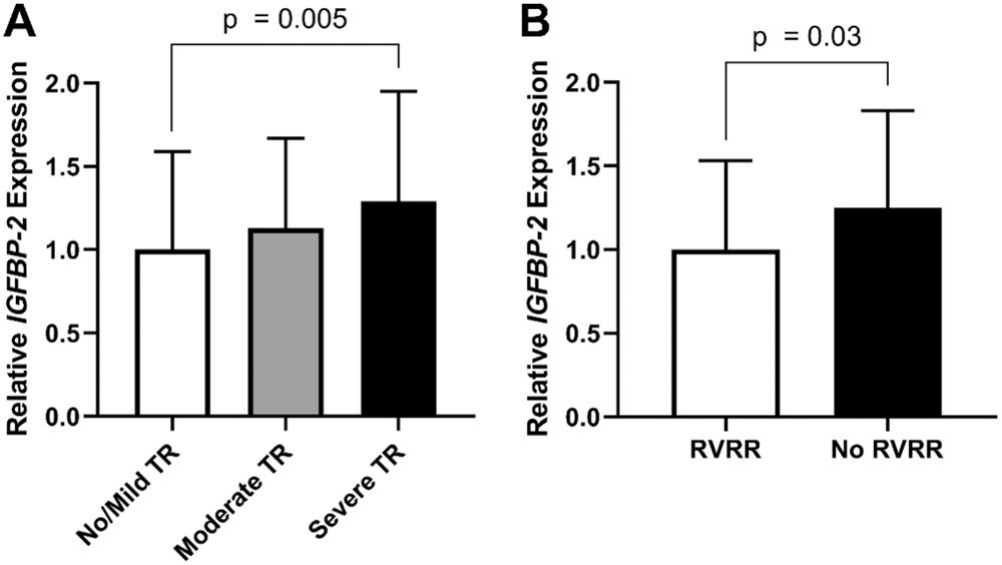
Normalized expression of IGFBP‐2 in patients with no/mild, moderate or severe concomitant TR (A) and in patients with and without right‐ventricular reverse remodeling within 3 months after M‐TEER (B). M‐TEER, mitral valve transcatheter edge‐to‐edge repair; RVRR, right‐ventricular reverse remodeling; TR, tricuspid regurgitation;

53 biomarkers were significantly regulated in patients with higher TR grades (Table 1). These included NT‐proBNP (upregulated 1.7‐fold in moderate TR and 2.4‐fold in severe TR compared to no/mild TR, *p* < 0.001), growth differentiation factor 15 (GDF‐15; 1.3‐fold and 1.6‐fold, *p* < 0.001), matrix metalloproteinase 2 (1.2‐fold and 1.4‐fold, *p* < 0.001) as well as insulin‐like growth factor binding proteins 1 (1.5‐fold and 1.9‐fold, *p* < 0.001), −7 (1.3‐fold and 1.7‐fold, *p* < 0.001) and −2. IGFBP‐2 was upregulated 1.2‐fold in patients with moderate TR and 1.4‐fold in patients with severe TR (*p* = 0.005) (Figure 1A).

**Table 1:** Biomarker expression in patients undergoing M‐TEER with no/mild, moderate and severe concomitant TR (NPX, base 2 logarithmic scale).

Biomarker	No/Mild TR (*n* = 94)		Moderate TR (*n* = 85)		Severe TR (*n* = 63)		*p*
	Mean	SD	Mean	SD	Mean	SD	
**TNFRSF‐14**	**5.46**	**0.95**	**5.66**	**0.82**	**5.81**	**0.83**	**0.004**
**LDL receptor**	**4.60**	**0.85**	**4.49**	**0.65**	**4.22**	**0.78**	**0.004**
ITGB‐2	6.44	0.58	6.44	0.43	6.52	0.59	0.59
**IL17‐RA**	**4.25**	**0.66**	**4.41**	**0.64**	**4.52**	**0.74**	**0.015**
**TNF‐R2**	**6.40**	**0.95**	**6.58**	**0.81**	**6.86**	**0.92**	**0.001**
MMP‐9	4.33	0.99	4.35	0.82	4.23	0.94	0.42
**EPHB‐4**	**5.70**	**0.70**	**5.83**	**0.58**	**5.97**	**0.66**	**0.006**
**IL2‐RA**	**5.02**	**0.85**	**5.14**	**0.80**	**5.33**	**0.82**	**0.044**
**OPG**	**4.40**	**0.74**	**4.57**	**0.57**	**4.67**	**0.59**	**0.001**
**ALCAM**	**8.26**	**0.61**	**8.33**	**0.37**	**8.48**	**0.57**	**0.002**
**TFF‐3**	**5.84**	**0.89**	**6.17**	**0.82**	**6.23**	**0.87**	**< 0.001**
SELP	9.79	0.70	9.76	0.48	9.72	0.71	0.85
**CSTB**	**4.99**	**1.12**	**5.35**	**0.97**	**5.49**	**1.06**	**< 0.001**
**MCP‐1**	**5.08**	**0.68**	**5.22**	**0.41**	**5.29**	**0.68**	**0.007**
**CD163**	**8.74**	**0.76**	**8.85**	**0.59**	**9.10**	**0.69**	**0.004**
Gal3	5.57	0.63	5.59	0.42	5.54	0.65	0.62
**GRN**	**6.23**	**0.54**	**6.33**	**0.40**	**6.51**	**0.55**	**< 0.001**
**NT‐proBNP**	**6.86**	**2.03**	**7.60**	**1.76**	**8.09**	**1.68**	**< 0.001**
BLM hydrolase	3.95	0.62	4.04	0.35	4.01	0.62	0.34
**PLC**	**9.36**	**0.56**	**9.55**	**0.50**	**9.62**	**0.52**	**< 0.001**
**LTBR**	**3.63**	**0.84**	**3.80**	**0.67**	**3.95**	**0.79**	**0.006**
**Notch 3**	**5.76**	**0.76**	**6.02**	**0.50**	**6.25**	**0.80**	**< 0.001**
TIMP‐4	5.08	0.78	5.22	0.69	5.18	0.62	0.08
CNTN‐1	3.47	0.70	3.53	0.48	3.67	0.70	0.12
**CDH‐5**	**4.77**	**0.68**	**4.81**	**0.45**	**4.97**	**0.64**	**0.026**
TLT‐2	5.35	0.77	5.37	0.62	5.41	0.72	0.69
**FABP‐4**	**5.85**	**1.48**	**6.35**	**1.35**	**6.23**	**1.49**	**0.027**
TFPI	10.10	0.73	10.20	0.64	10.08	0.61	0.25
PAI	4.95	1.09	5.28	1.21	5.31	1.26	0.08
CCL‐24	5.83	1.19	6.02	1.11	5.72	1.18	0.13
**TR**	**6.90**	**0.83**	**7.19**	**0.79**	**7.23**	**0.98**	**0.028**
TNFRSF‐10C	5.84	0.63	5.83	0.61	5.91	0.71	0.71
**GDF‐15**	**6.51**	**1.02**	**6.90**	**0.95**	**7.15**	**1.04**	**< 0.001**
SELE	12.06	0.72	12.03	0.62	11.97	0.84	0.90
AZU‐1	3.18	0.95	3.40	1.05	3.12	0.77	0.18
DLK‐1	7.55	1.00	7.53	0.87	7.61	0.96	0.87
**SPON‐1**	**1.55**	**1.00**	**1.78**	**0.99**	**1.70**	**0.73**	**0.003**
MPO	3.91	0.64	3.96	0.63	3.84	0.60	0.58
**CXCL‐16**	**6.65**	**0.65**	**6.76**	**0.38**	**6.86**	**0.66**	**0.013**
IL6‐RA	13.40	0.56	13.41	0.45	13.38	0.53	0.48
RETN	7.34	0.87	7.37	0.72	7.44	0.78	0.47
**IGFPB‐1**	**7.72**	**1.18**	**8.34**	**1.10**	**8.61**	**1.13**	**< 0.001**
CHIT‐1	5.27	2.44	5.44	2.22	5.56	2.41	0.43
**TRAP**	**5.05**	**0.69**	**4.85**	**0.52**	**4.76**	**0.63**	**0.001**
GP‐6	1.74	0.61	1.81	0.53	1.93	0.63	0.07
PSPD	3.77	0.96	3.70	0.86	3.81	0.95	0.98
PI‐3	3.64	0.99	3.84	1.07	3.83	0.97	0.18
EpCAM	5.32	1.11	5.08	1.01	5.25	1.08	0.43
**APN**	**6.90**	**0.59**	**7.04**	**0.51**	**7.29**	**0.61**	**< 0.001**
**AXL**	**9.11**	**0.68**	**9.28**	**0.48**	**9.52**	**0.60**	**< 0.001**
**IL1‐RT1**	**5.84**	**0.71**	**5.94**	**0.42**	**6.15**	**0.64**	**0.003**
**MMP‐2**	**3.42**	**0.73**	**3.65**	**0.54**	**3.87**	**0.64**	**< 0.001**
**FAS**	**6.77**	**0.68**	**6.89**	**0.50**	**7.04**	**0.68**	**0.003**
MB	8.53	0.95	8.71	0.80	8.78	1.04	0.10
**TNFSF‐13B**	**7.61**	**0.72**	**7.65**	**0.58**	**7.92**	**0.90**	**0.039**
PRTN‐3	4.30	0.71	4.45	0.65	4.37	0.73	0.15
PCSK‐9	3.06	0.64	3.16	0.53	3.04	0.69	0.06
**UPAR**	**6.58**	**0.81**	**6.79**	**0.63**	**6.95**	**0.69**	**< 0.001**
**OPN**	**8.72**	**1.03**	**9.06**	**0.85**	**9.16**	**0.79**	**0.001**
**CTSD**	**4.23**	**0.57**	**4.44**	**0.53**	**4.57**	**0.64**	**< 0.001**
PGLYRP‐1	8.56	0.79	8.54	0.73	8.64	0.66	0.51
CPA‐1	6.71	1.10	6.78	0.97	7.05	1.08	0.06
**JAMA**	**5.80**	**0.94**	**6.07**	**0.73**	**6.36**	**0.89**	**< 0.001**
Gal4	5.18	0.77	5.20	0.69	5.31	0.77	0.43
IL1‐RT2	6.47	0.61	6.49	0.45	6.64	0.62	0.11
**SHPS‐1**	**4.08**	**0.80**	**4.17**	**0.58**	**4.38**	**0.62**	**0.013**
**CCL‐15**	**8.98**	**0.80**	**9.18**	**0.65**	**9.24**	**0.67**	**0.004**
CASP‐3	4.41	0.72	4.53	0.63	4.51	0.87	0.26
**uPA**	**5.53**	**0.60**	**5.58**	**0.37**	**5.74**	**0.63**	**0.028**
CPB‐1	7.53	1.00	7.65	0.87	7.79	0.95	0.14
**CHI3L1**	**7.50**	**1.18**	**7.85**	**1.11**	**7.84**	**1.03**	**0.046**
**ST‐2**	**5.53**	**0.92**	**5.70**	**0.86**	**6.11**	**0.98**	**< 0.001**
tPA	6.06	0.84	6.20	0.81	6.16	0.92	0.16
**SCGB3A2**	**3.03**	**1.08**	**3.51**	**1.31**	**3.58**	**0.82**	**< 0.001**
EGFR	3.47	0.53	3.44	0.24	3.47	0.51	0.94
**IGFBP‐7**	**8.76**	**1.02**	**9.10**	**0.68**	**9.48**	**0.99**	**< 0.001**
**CD93**	**11.64**	**0.58**	**11.80**	**0.47**	**11.89**	**0.49**	**0.009**
**IL18‐BP**	**7.36**	**0.69**	**7.49**	**0.57**	**7.64**	**0.63**	**0.01**
**COL1A1**	**2.69**	**0.65**	**2.95**	**0.61**	**3.03**	**0.64**	**0.001**
**PON‐3**	**5.73**	**0.90**	**5.54**	**0.71**	**5.35**	**0.96**	**0.006**
**CTSZ**	**5.41**	**0.54**	**5.57**	**0.53**	**5.57**	**0.55**	**0.025**
**MMP‐3**	**6.82**	**1.05**	**7.03**	**0.95**	**7.13**	**0.99**	**0.041**
RARRES‐2	11.93	0.39	11.99	0.34	11.88	0.39	0.07
**ICAM‐2**	**5.98**	**0.66**	**6.07**	**0.37**	**6.21**	**0.63**	**0.02**
KLK‐6	5.05	0.81	4.98	0.60	5.01	0.67	0.95
PDGFsub‐A	2.06	1.23	2.02	1.10	2.04	1.07	0.94
**TNF‐R1**	**7.97**	**0.96**	**8.21**	**0.85**	**8.42**	**0.89**	**< 0.001**
**IGFBP‐2**	**9.66**	**0.84**	**9.91**	**0.73**	**10.10**	**0.71**	**0.004**
vWF	6.70	1.14	6.84	0.93	7.05	1.05	0.07
**PECAM‐1**	**4.90**	**0.56**	**4.93**	**0.42**	**5.07**	**0.59**	**0.03**
MEPE	4.87	0.90	4.99	0.73	5.00	0.74	0.22
**CCL‐16**	**8.07**	**0.81**	**8.23**	**0.59**	**8.12**	**0.74**	**0.045**

*Note:* Statistically significant differences are marked in bold.

Abbreviations: M‐TEER, mitral valve transcatheter edge‐to‐edge repair; NPX, normalized protein expression; SD, standard deviatabletion; TR, tricuspid regurgitation.

### 3.2 Three‐Month Course of TR and RV Diameters After M‐TEER

A complete echocardiographic assessment 3 months after M‐TEER could be carried out in 99 patients with at least moderate concomitant TR at baseline. RVRR as a combined endpoint occurred in 50 patients (50.5%) while it was not registered in 49 patients (49.5%). The main driver of this endpoint was TR reduction, which was seen in 46 patients (46.5%), while 15 patients (15.2%) had RV diameter downsizing of at least 10%.

### 3.3 Clinical Determinants of RVRR

Patients, which did not develop RVRR had a significantly higher prevalence of chronic pulmonary disease. Furthermore, etiology of MR was classified as degenerative in a majority of patients without RVRR, whereas in the RVRR group more patients were classified as having functional MR. The procedural success rate was considerably lower in patients without RVRR, however this difference was statistically insignificant (Table 2).

**Table 2:** Baseline Characteristics of patients with and without right‐ventricular reverse remodeling within 3 months after M‐TEER.

Variable	RVRR (*n* = 50)	No RVRR (*n* = 49)	*p*
Age (years)	80.0 (73.0–82.3)	80.0 (76.0–82.5)	0.52
Female Sex	20/50 (40.0%)	19/49 (38.8%)	0.90
BMI (kg/m²)	25.4 ± 4.5	25.6 ± 4.0	0.82
*Baseline NYHA‐class*			0.42
II	11/50 (22.0%)	6/49 (12.2%)	
III	30/50 (60.0%)	32/49 (65.3%)	
IV	9/50 (18.0%)	11/49 (22.4%)	
*Baseline MR‐grade*			0.36
III	13/50 (26.0%)	9/49 (18.4%)	
IV	37/50 (74.0%)	40/49 (81.6%)	
*MR etiology*			0.06
Degenerative	11/50 (22.0%)	21/49 (43.8%)	
Functional	29/50 (58.0%)	18/49 (37.5%)	
Mixed	10/50 (20.0%)	9/49 (18.8%)	
Procedural Success	47/50 (94.0%)	41/49 (85.4%)	0.16
*MR‐grade at discharge*			0.13
< I	10/50 (20.0%)	4/49 (8.3%)	
I	23/50 (46.0%)	22/49 (45.8%)	
II	15/50 (30.0%)	16/49 (33.3%)	
III	1/50 (2.0%)	6/49 (12.5%)	
IV	1/50 (2.0%)	0	
MV‐Gradient post TEER (mmHg)	3.0 (2.0–5.0)	4.0 (3.0–5.0)	0.36
EuroSCORE II	5.3 (3.0–8.0)	5.1 (3.4–9.7)	0.82
STS‐Score	3.7 (2.3–6.6)	3.4 (2.3–8.4)	0.55
CAD	33/50 (66.0%)	28/49 (57.1%)	0.37
DCM	9/50 (18.0%)	7/49 (14.3%)	0.62
Atrial Fibrillation	27/50 (54.0%)	28/49 (57.1%)	0.75
**Pulmonary Disease**	**1/50 (2.0%)**	**13/49 (26.5%)**	**< 0.001**
Diabetes mellitus	10/50 (20.0%)	11/49 (22.4%)	0.77
Renal Failure	27/50 (54.0%)	27/49 (55.1%)	0.91
ACE‐Inhibitor/AT1‐Blocker	37/50 (74.0%)	33/49 (67.3%)	0.47
ARNI	7/50 (14.0%)	7/49 (14.3%)	0.97
Betablocker	44/50 (88.0%)	43/49 (87.8%)	0.97
Aldosterone antagonist	30/50 (60.0%)	28/49 (57.1%)	0.77
SGLT2‐Inhibitor	1 (2.0%)	0	0.32
Loop diuretics	43/50 (86.0%)	43/49 (87.8%)	0.80
Creatinine (µmol/L)	124.5 (97.8–156.3)	124.0 (91.5–158.0)	0.87
eGFR (mL/min/1.73 m²)	45.5 ± 17.4	45.3 ± 18.9	0.95
Troponin T (ng/L)	32.0 (17.5–40.0)	30.0 (18.0–45.0)	0.77
NT‐proBNP (pg/mL)	2707.5 (1351.5–6123.8)	5107.0 (2639.0–7778.0)	0.06
Hb (g/dL)	12.7 (11.8–14.2)	12.8 (11.1–13.8)	0.54
sPAP (mmHg)	55.0 ± 15.4	59.1 ± 16.0	0.21
TAPSE (mm)	18.8 ± 5.1	18.3 ± 5.0	0.66
TAPSE/sPAP ratio	0.31 (0.23–0.48)	0.29 (0.23–0.46)	0.26
*LV‐EF*			0.38
> 50%	18/50 (36.0%)	19/49 (38.8%)	
41−49%	7/50 (14.0%)	11/49 (22.4%)	
31−40%	10/50 (20.0%)	11/49 (22.4%)	
< 30%	15/50 (30.0%)	8/49 (16.3%)	
LVEDD (mm)	60.3 ± 10.0	58.5 ± 9.3	0.34
Basal RV diameter (mm)	48.0 (43.0–54.5)	49.5 (45.0–54.8)	0.53
*TR‐Severity*			0.91
Moderate	28/50 (56.0%)	28/49 (57.1%)	
Severe	22/50 (44.0%)	21/49 (42.9%)	

*Note:* Statistically significant differences are marked in bold.

Abbreviations: ARNI, angiotensin‐receptor‐neprilysin‐inhibitor; BMI, body mass index; CAD, coronary artery disease; DCM, dilatative cardiomyopathy; eGFR, estimated glomerular filtration rate; Hb, hemoglobin; LVEDD, left‐ventricular end‐diastolic diameter; LV‐EF, left‐ventricular ejection fraction; MR, mitral regurgitation; M‐TEER, mitral valve transcatheter edge‐to‐edge repair; MV, mitral valve; NYHA, New York Heart Association; RV, right ventricle; RVRR, right‐ventricular reverse remodeling; sPAP, systolic pulmonary artery pressure; TAPSE, tricuspid anular plane systolic excursion; TR, tricuspid regurgitation.

Significant differential regulation was seen only for one biomarker: IGFBP‐2 was upregulated 1.3‐fold in patients without RVRR (*p* = 0.03) (Supporting Information S1: Table 2 and Figure 1B). Upregulation of IGFBP‐7 was borderline significant, however this effect did not persist after linearization of the logarithmic scale.

**Figure 2:**
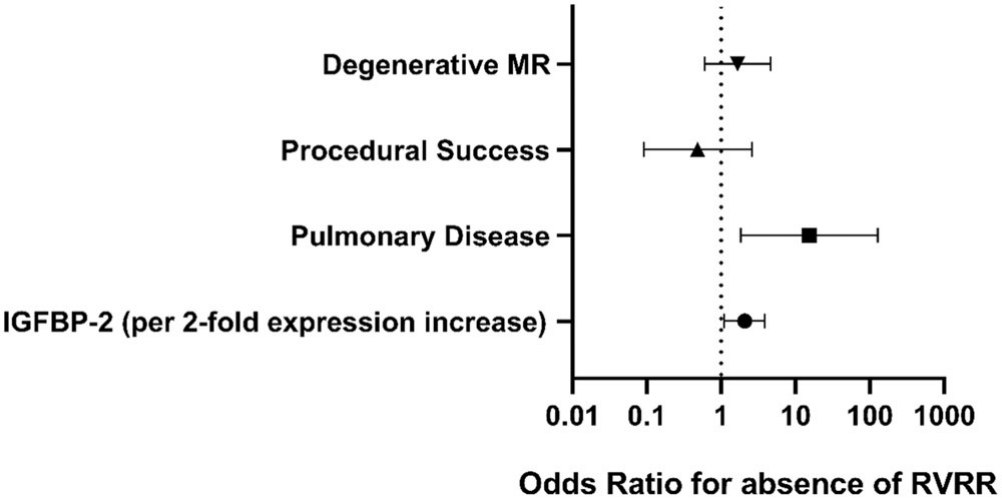
Forest plot of logistic regression for prediction of absence of right‐ventricular reverse remodeling within 3 months after M‐TEER. MR, mitral regurgitation; M‐TEER, mitral valve transcatheter edge‐to‐edge repair; RVRR, right‐ventricular reverse remodeling.

After adjustment for covariates, two factors were independently associated with absence of RVRR 3 months after M‐TEER: higher expression levels of IGFBP‐2 predicted persistent TR or RV dilation with an Odds Ratio (OR) of 2.078 (95% confidence interval (CI) 1.108−3.897, *p* = 0.023). Presence of chronic pulmonary disease was an even stronger predictor of absence of RVRR (OR 15.341, 95% CI 1.838−128.063, *p* = 0.012) (Supporting Information S1: Table 3 and Figure 2). Since NT‐proBNP correlated significantly with IGFBP‐2 it was excluded from the initial multivariate analysis. In a separate analysis including NT‐proBNP and excluding IGFBP‐2, NT‐proBNP showed no association with RVRR (Supporting Information S1: Table 4). No relevant difference was found regarding IGFBP‐2 expression in patients with and without chronic pulmonary disease (mean expression 9.9 ± 0.8 vs. 9.9 ± 0.8 NPX, *p* = 0.91).

## 4 Discussion

Concomitant RV remodeling and significant functional TR are common comorbidities in patients undergoing M‐TEER. Persistent TR after M‐TEER is associated with worse mid‐ or long‐term outcome, especially in functional MR [10, 11]. Early staged or even simultaneous tricuspid valve repair is a therapeutic option in these patients [12, 13]. Accurate prediction of the TR course after M‐TEER is important for adequate post‐procedural management of these high‐risk patients, especially within 3 months following mitral valve repair, as TR improvement occurs mainly during this period [3, 4]. Certain clinical factors have been identified, that are associated with secondary regression of concomitant TR after M‐TEER [6, 7]. However, no data exist on the usability of serum biomarkers for prediction of TR course.

In this prospective study, we identified a large number of cardiometabolic biomarkers, that are upregulated in MR patients with concomitant TR. One of the most significantly regulated markers, NT‐proBNP, is the most prominently used biomarker for diagnosis and risk prediction in heart failure [14]. Specific data on patients with RV failure are sparse, however in patients with precapillary pulmonary hypertension (PH) NT‐proBNP correlates with RV strain, is associated with adverse outcome and is therefore part of the Mayo clinic right heart score [15]. On the other hand, data from acutely hospitalized HF patients suggest poor prognostic value of NT‐proBNP regarding mortality, when severe TR is present [16]. Efforts to find specific biomarkers for RV failure in the context of PH have identified peptides like SPARC‐like protein 1 (SPARCL1) or cartilage intermediate layer protein 1 (CILP1) but also long non‐coding RNA H19 [17]. Specifically in HF patients with RV dysfunction, Galectin‐3 correlated negatively with RV function and exercise capacity [18]. None of these markers however have yet been introduced into clinical practice. Our study provides numerous new candidates in a HF cohort with advanced RV dilation and TR, however individual characterization of each biomarker is needed.

Specifically, three subtypes of the insulin‐like growth factor binding protein‐family (IGFBP) were upregulated in patients with moderate or severe TR: IGFBP‐1, ‐2 and ‐7. Robust data link both IGFBP‐2 and ‐7 to higher risk for adverse events in HF patients and show an incremental effect to clinical variables and NT‐proBNP [19, 20]. Our data now show, that both proteins are upregulated even further in HF patients with significant concomitant TR compared to HF patients without TR. While the mechanism of action of IGFBP subtypes is a matter of ongoing research, previous work has shown that IGFBP‐7 promotes cardiomyocyte senescence and mediates inflammatory processes in patients with heart failure with preserved ejection fraction (HFpEF) [21, 22].

Our study is the first to identify a serum biomarker predicting the course of RVRR after M‐TEER. Higher levels of IGFBP‐2 predicted non‐improvement of TR and/or absence of significant RV diameter reduction. Furthermore, presence of chronic pulmonary disease was identified as an even stronger predictor. Importantly however, IGFBP‐2 was not associated with pulmonary disease. It can be proposed that higher expression levels of IGFBP subtypes and specifically IGFBP‐2 indicate advanced and irreversible stages of RV remodeling with persistent TR even after alleviation of RV afterload by mitral valve repair. Use of this biomarker in patients with bivalvular regurgitation could be useful to guide clinical management and identify patients in need for early tricuspid valve repair. However, given the moderate sample size causality cannot yet be inferred and further studies are needed to validate our results.

Chronic pulmonary disease strongly predicted RVRR non‐development in our study. Pulmonary diseases can lead to significant precapillary PH. In this case, reduction of postcapillary pressure components through M‐TEER might result in insufficient relief in RV afterload, thus causing persistence of TR. A worse outcome of patients with pulmonary comorbidities undergoing M‐TEER has been reported previously [23].

### 4.1 Study Limitations

While our data were obtained prospectively, the number of included patients is only moderate. Especially, the high dropout rate before the 3‐month follow‐up must be emphasized, which reduces statistical power and increases the risk of selection bias. The main reason was the emerging COVID‐19 pandemic, which led to restrictions in follow‐up visits. Second, due to the cohort size we cannot reasonably differentiate between primary and secondary MR etiology. This however implies an “all‐comers” cohort for routine practice. Lastly, we report data from a monocentric cohort study. Hence, it needs to be stressed that our results can only be appreciated as hypothesis‐generating. External validation in larger, multicentric collectives is needed to implement these results into clinical practice.

## 5 Conclusions

In patients undergoing M‐TEER with RV dilation and concomitant moderate or severe TR, numerous cardiometabolic biomarkers including NT‐proBNP, IGFBP‐2 and IGFBP‐7 are upregulated. Aside from chronic pulmonary disease, higher levels of IGFBP‐2 at baseline are independently associated with persistent TR and/or RV dilation after M‐TEER. This hypothesis‐generating study warrants further examination of this biomarker in the context of bivalvular regurgitation.

## Ethics Statement

The study received ethical oversight from the ethics committee of the University of Ulm.

## Conflicts of Interest

The authors declare no conflicts of interest.

## Supporting information

Supporting information.

## Data Availability

The data will be made available upon reasonable request.
