# Supplementary material for: Insulin‐Like Growth Factor Binding Protein 2 Predicts Right Ventricular Reverse Remodeling and Improvement of Concomitant Tricuspid Regurgitation After Transcatheter Edge‐to‐Edge Mitral Valve Repair
Source: Clin Cardiol. 2024 Nov 26;47(12):e70048. doi: 10.1002/clc.70048 (PMC11599423; doi:10.1002/clc.70048)
Supplement: Supplementary file 1 — Supporting information. [file CLC-47-e70048-s001.docx]

**Supplemental Material for Manuscript**

**Insulin-like growth factor binding protein 2 predicts right ventricular reverse remodeling and improvement of concomitant tricuspid regurgitation after transcatheter edge-to-edge mitral valve repair**

**by Matthias Gröger et al.**

Supplemental Table 1: Baseline Characteristics of patients undergoing M-TEER with no/mild, moderate and severe concomitant TR

| **Variable** | **No/Mild TR**  **(n = 94)** | **Moderate TR**  **(n = 85)** | **Severe TR**  **(n = 63)** | **p** |
| --- | --- | --- | --- | --- |
| **Age (years)** | **77.0 (71.0 – 80.0)** | **80.0 (73.0 – 83.5)** | **81.0 (76.0 – 83.0)** | **0.003** |
| Female Sex | 39/94 (41.5%) | 41/85 (48.2%) | 27/63 (42.9%) | 0.64 |
| BMI (kg/m²) | 26.4 ± 4.6 | 26.0 ± 4.8 | 25.9 ± 5.1 | 0.65 |
| Baseline NYHA-Class  II  III  IV | 21/94 (22.3%)  58/94 (61.7%)  15/94 (16.0%) | 13/85 (15.3%)  52/85 (61.2%)  20/85 (23.5%) | 10/63 (15.9%)  44/63 (69.8%)  9/63 (14.3%) | 0.39 |
| Baseline MR-Grade  III  IV | 20/94 (21.3%)  74/94 (78.7%) | 12/85 (14.1%)  73/85 (85.9%) | 15/63 (23.8%)  48/63 (76.2%) | 0.29 |
| MR Etiology  Degenerative  Functional  Mixed | 39/94 (41.9%)  37/94 (39.8%)  17/94 (18.3%) | 36/85 (42.4%)  31/85 (36.5%)  18/85 (21.2%) | 23/63 (37.1%)  29/63 (46.8%) 10/63 (16.1%) | 0.78 |
| Procedural Success | 85/94 (90.4%) | 73/85 (85.9%) | 58/63 (92.0%) | 0.26 |
| MR-Grade at Discharge  <I  I  II  III  IV | 21/94 (22.6%)  32/94 (34.4%)  34/94 (36.6%)  6/94 (6.5%)  0 | 14/85 (16.5%)  30/85 (35.3%)  31/85 (36.5%)  8/85 (9.4%)  2/85 (2.4%) | 6/63 (9.7%)  30/63 (48.4%)  23/63 (37.1%)  3/63 (4.8%)  0 | 0.22 |
| MV-Gradient post TEER (mmHg) | 3.0 (2.0 – 5.0) | 4.0 (3.0 – 5.0) | 3.0 (2.0 – 5.0) | 0.80 |
| **EuroSCORE II** | **3.2 (2.2 – 6.2)** | **4.9 (3.0 – 8.0)** | **5.2 (3.5 – 10.2)** | **0.002** |
| **STS-Score** | **2.5 (1.4 – 4.4)** | **3.2 (2.1 – 5.8)** | **4.5 (2.7 – 8.6)** | **< 0.001** |
| **CAD** | **62/94 (66.0%)** | **57/85 (67.1%)** | **28/63 (44.4%)** | **0.009** |
| DCM | 17/94 (18.1%) | 13/85 (15.3%) | 13/63 (20.6%) | 0.70 |
| Atrial Fibrillation | 51/94 (54.3%) | 48/85 (56.5%) | 41/63 (65.1%) | 0.38 |
| Pulmonary Disease | 16/94 (17.0%) | 13/85 (15.3%) | 9/63 (14.3%) | 0.89 |
| Diabetes mellitus | 32/94 (34.0%) | 19/85 (22.4%) | 14/63 (22.2%) | 0.13 |
| Renal Failure | 40/94 (42.6%) | 45/85 (52.9%) | 36/63 (57.1%) | 0.16 |
| **ACE-Inhibitor/ AT1-Blocker** | **75/94 (79.8%)** | **63/85 (74.1%)** | **36/63 (57.1%)** | **0.007** |
| ARNI | 10/94 (10.6%) | 13/85 (15.3%) | 7/63 (11.1%) | 0.60 |
| Betablocker | 81/94 (86.2%) | 78/85 (91.8%) | 53/63 (84.1%) | 0.33 |
| Aldosterone antagonist | 45/94 (47.9%) | 45/85 (52.9%) | 32/63 (50.8%) | 0.79 |
| SGLT2-Inhibitor | 1/94 (1.1%) | 1/85 (1.2%) | 0 | 0.70 |
| Loop diuretics | 66/94 (70.2%) | 71(85 (83.5%) | 52/63 (82.5%) | 0.06 |
| **Creatinine (µmol/l)** | **104.5 (90.0 – 137.3)** | **120.0 (97.5 – 155.0)** | **130.0 (92.0 – 183.0)** | **0.024** |
| **eGFR (ml/min/1,73 m²)** | **52.1 ± 19.7** | **46.1 ± 17.8** | **47.6 ± 19.4** | **0.008** |
| **Troponin T (ng/l)** | **21.0 (16.0 – 34.0)** | **28.5 (17.0 – 43.0)** | **33.0 (21.0 – 47.8)** | **0.011** |
| **NT-proBNP (pg/ml)** | **1770.0 (874.0 – 3714.0)** | **3745.0 (1519.5 – 7080.8)** | **4335.0 (2384.5 – 8154.0)** | **< 0.001** |
| Hb (g/dl) | 12.9 (11.5 – 14.0) | 12.5 (11.6 – 13.9) | 12.5 (10.7 – 13.9) | 0.44 |
| sPAP (mmHg) | 51.0 ± 17.8 | 54.3 ± 15.5 | 50.5 ± 15.9 | 0.16 |
| LV-EF  >50%  41-49%  31-40%  <30% | 37/94 (39.4%)  21/94 (22.3%)  23/94 (24.5%)  13/94 (13.8%) | 32/85 (37.6%)  15/85 (17.6%)  18/85 (21.2%)  20/85 (23.5%) | 31/63 (49.2%)  11/63 (17.5%)  8/63 (12.7%)  13/63 (20.6%) | 0.33 |
| **LVEDD (mm)** | **61.5 ± 11.4** | **61.0 ± 10.9** | **60.1 ± 11.2** | **0.019** |
| **Basal RV Diameter (mm)** | **43.5 (38.0 – 48.0)** | **46.0 (42.8 – 51.0)** | **53.5 (46.0 – 59.3)** | **< 0.001** |

*M-TEER: mitral valve transcatheter edge-to-edge repair; TR: tricuspid regurgitation; BMI: body mass index; NYHA: New York Heart Association; MR: mitral regurgitation; MV: mitral valve; CAD: coronary artery disease; DCM: dilatative cardiomyopathy; ARNI: angiotensin-receptor-neprilysin-inhibitor; eGFR: estimated glomerular filtration rate; Hb: hemoglobin; sPAP: systolic pulmonary artery pressure; LV-EF: left-ventricular ejection fraction; LVEDD: left-ventricular end-diastolic diameter; RV: right ventricle*

Supplemental Table 2: Biomarker expression in patients with and without right-ventricular reverse remodeling within 3 months after M-TEER (base 2 logarithmic scale)

| **Biomarker** | **RVRR**  **(n = 50)** | | **No RVRR**  **(n = 49)** | | **p** |
| --- | --- | --- | --- | --- | --- |
|  | **Mean** | **SD** | **Mean** | **SD** |  |
| TNFRSF-14 | 5.64 | 0.82 | 5.75 | 0.82 | 0.50 |
| LDL receptor | 4.52 | 0.82 | 4.26 | 0.66 | 0.08 |
| ITGB-2 | 6.54 | 0.54 | 6.43 | 0.54 | 0.30 |
| IL17-RA | 4.42 | 0.69 | 4.45 | 0.71 | 0.87 |
| TNF-R2 | 6.66 | 0.90 | 6.68 | 0.88 | 0.91 |
| MMP-9 | 4.27 | 0.92 | 4.47 | 0.92 | 0.29 |
| EPHB-4 | 5.79 | 0.65 | 5.92 | 0.62 | 0.32 |
| IL2-RA | 5.20 | 0.94 | 5.27 | 0.85 | 0.72 |
| OPG | 4.59 | 0.61 | 4.71 | 0.56 | 0.32 |
| ALCAM | 8.39 | 0.53 | 8.41 | 0.48 | 0.86 |
| TFF-3 | 6.09 | 0.79 | 6.28 | 0.89 | 0.28 |
| SELP | 9.87 | 0.63 | 9.80 | 0.61 | 0.57 |
| CSTB | 5.28 | 0.99 | 5.47 | 1.01 | 0.34 |
| MCP-1 | 5.24 | 0.58 | 5.34 | 0.53 | 0.36 |
| CD163 | 9.03 | 0.64 | 8.94 | 0.66 | 0.48 |
| Gal3 | 5.63 | 0.58 | 5.61 | 0.53 | 0.90 |
| GRN | 6.39 | 0.50 | 6.43 | 0.52 | 0.67 |
| NT-proBNP | 7.60 | 1.75 | 7.91 | 1.77 | 0.39 |
| BLM hydrolase | 4.08 | 0.51 | 4.03 | 0.51 | 0.61 |
| PLC | 9.53 | 0.52 | 9.65 | 0.55 | 0.24 |
| LTBR | 3.76 | 0.76 | 3.90 | 0.70 | 0.34 |
| Notch 3 | 6.04 | 0.73 | 6.17 | 0.68 | 0.36 |
| TIMP-4 | 5.14 | 0.68 | 5.28 | 0.75 | 0.33 |
| CNTN-1 | 3.61 | 0.66 | 3.65 | 0.59 | 0.75 |
| CDH-5 | 4.85 | 0.63 | 4.95 | 0.52 | 0.40 |
| TLT-2 | 5.36 | 0.76 | 5.39 | 0.69 | 0.87 |
| FABP-4 | 6.08 | 1.20 | 6.25 | 1.53 | 0.53 |
| TFPI | 10.08 | 0.62 | 10.30 | 0.77 | 0.13 |
| PAI | 5.28 | 1.11 | 5.38 | 1.46 | 0.69 |
| CCL-24 | 6.07 | 1.11 | 5.83 | 1.14 | 0.29 |
| TR | 7.10 | 0.78 | 7.06 | 0.95 | 0.83 |
| TNFRSF-10C | 5.83 | 0.71 | 5.91 | 0.67 | 0.56 |
| GDF-15 | 6.91 | 0.95 | 7.08 | 1.10 | 0.40 |
| SELE | 12.08 | 0.72 | 12.05 | 0.71 | 0.83 |
| AZU-1 | 3.29 | 0.81 | 3.51 | 1.25 | 0.30 |
| DLK-1 | 7.49 | 0.90 | 7.69 | 0.95 | 0.30 |
| SPON-1 | 1.59 | 0.77 | 1.72 | 1.01 | 0.28 |
| MPO | 3.88 | 0.59 | 4.05 | 0.74 | 0.20 |
| CXCL-16 | 6.71 | 0.52 | 6.81 | 0.46 | 0.27 |
| IL6-RA | 13.44 | 0.53 | 13.48 | 0.52 | 0.75 |
| RETN | 7.36 | 0.72 | 7.40 | 0.71 | 0.82 |
| IGFPB-1 | 8.32 | 1.20 | 8.62 | 0.96 | 0.17 |
| CHIT-1 | 5.34 | 2.65 | 5.68 | 2.01 | 0.47 |
| TRAP | 4.91 | 0.62 | 4.79 | 0.59 | 0.33 |
| GP-6 | 1.82 | 0.62 | 1.88 | 0.55 | 0.60 |
| PSPD | 3.87 | 0.99 | 3.77 | 0.85 | 0.62 |
| PI-3 | 3.83 | 1.03 | 3.86 | 0.93 | 0.87 |
| EpCAM | 5.13 | 0.96 | 5.39 | 1.15 | 0.23 |
| APN | 7.12 | 0.58 | 7.19 | 0.53 | 0.57 |
| AXL | 9.37 | 0.58 | 9.33 | 0.54 | 0.73 |
| IL1-RT1 | 5.98 | 0.61 | 6.06 | 0.47 | 0.46 |
| MMP-2 | 3.67 | 0.68 | 3.84 | 0.53 | 0.15 |
| FAS | 6.95 | 0.70 | 7.01 | 0.58 | 0.67 |
| MB | 8.75 | 0.96 | 8.83 | 0.91 | 0.69 |
| TNFSF-13B | 7.67 | 0.68 | 7.82 | 0.83 | 0.33 |
| PRTN-3 | 4.50 | 0.79 | 4.34 | 0.64 | 0.27 |
| PCSK-9 | 3.11 | 0.72 | 3.15 | 0.57 | 0.80 |
| UPAR | 6.81 | 0.68 | 6.88 | 0.65 | 0.65 |
| OPN | 8.99 | 0.78 | 9.12 | 0.86 | 0.42 |
| CTSD | 4.44 | 0.56 | 4.59 | 0.61 | 0.23 |
| PGLYRP-1 | 8.57 | 0.70 | 8.62 | 0.74 | 0.71 |
| CPA-1 | 6.98 | 0.89 | 6.96 | 1.00 | 0.94 |
| JAMA | 6.08 | 0.78 | 6.31 | 0.81 | 0.15 |
| Gal4 | 5.14 | 0.76 | 5.38 | 0.71 | 0.11 |
| IL1-RT2 | 6.60 | 0.57 | 6.52 | 0.54 | 0.47 |
| SHPS-1 | 4.21 | 0.64 | 4.24 | 0.64 | 0.82 |
| CCL-15 | 9.24 | 0.67 | 9.20 | 0.62 | 0.79 |
| CASP-3 | 4.58 | 0.84 | 4.68 | 0.78 | 0.52 |
| uPA | 5.68 | 0.54 | 5.65 | 0.51 | 0.76 |
| CPB-1 | 7.80 | 0.83 | 7.65 | 0.96 | 0.42 |
| CHI3L1 | 7.78 | 1.05 | 7.88 | 1.13 | 0.65 |
| ST-2 | 5.78 | 0.98 | 5.95 | 0.90 | 0.40 |
| tPA | 6.35 | 1.10 | 6.20 | 0.76 | 0.42 |
| SCGB3A2 | 3.63 | 0.89 | 3.44 | 1.10 | 0.37 |
| EGFR | 3.49 | 0.49 | 3.45 | 0.36 | 0.68 |
| IGFBP-7 | 9.10 | 0.89 | 9.43 | 0.79 | 0.05 |
| CD93 | 11.77 | 0.51 | 11.87 | 0.48 | 0.29 |
| IL18-BP | 7.54 | 0.62 | 7.53 | 0.62 | 0.97 |
| COL1A1 | 2.88 | 0.63 | 3.03 | 0.63 | 0.25 |
| PON-3 | 5.46 | 0.94 | 5.53 | 0.75 | 0.69 |
| CTSZ | 5.58 | 0.53 | 5.59 | 0.56 | 0.96 |
| MMP-3 | 7.09 | 1.03 | 7.16 | 0.97 | 0.74 |
| RARRES-2 | 11.93 | 0.39 | 11.97 | 0.38 | 0.54 |
| ICAM-2 | 6.07 | 0.58 | 6.17 | 0.50 | 0.35 |
| KLK-6 | 5.06 | 0.68 | 4.98 | 0.58 | 0.57 |
| PDGFsub-A | 2.02 | 1.06 | 2.21 | 1.30 | 0.43 |
| TNF-R1 | 8.23 | 0.85 | 8.34 | 0.87 | 0.53 |
| **IGFBP-2** | **9.80** | **0.75** | **10.14** | **0.72** | **0.022** |
| vWF | 6.95 | 1.08 | 7.05 | 0.90 | 0.62 |
| PECAM-1 | 5.02 | 0.54 | 5.05 | 0.53 | 0.79 |
| MEPE | 5.05 | 0.79 | 5.04 | 0.72 | 0.94 |
| CCL-16 | 8.13 | 0.76 | 8.23 | 0.63 | 0.51 |

*M-TEER: mitral valve transcatheter edge-to-edge repair; TR: tricuspid regurgitation;
NPX: normalized protein expression; SD: standard deviation*

Supplemental Table 3: Logistic regression for prediction of absence of right-ventricular reverse remodeling within 3 months after M-TEER

|  | Univariate Logistic Regression | | | Multivariate Logistic Regression | | | |
| --- | --- | --- | --- | --- | --- | --- | --- |
|  | Odds Ratio | 95% CI | p | Odds Ratio | 95% CI | p |  |
| **IGFBP-2** | **1.918** | **1.083 – 3.397** | **0.026** | **2.078** | **1.108 – 3.897** | **0.023** |  |
| Procedural Success | 0.374 | 0.091 – 1.540 | 0.173 | 0.485 | 0.091 – 2.592 | 0.397 |  |
| NT-proBNP  (per 1,000 pg/ml) | 1.042 | 0.981 – 1.106 | 0.179 | excluded due to correlation with IGFBP-2 | | | |
| **Pulmonary Disease** | **17.694** | **2.213 – 141.487** | **0.007** | **15.341** | **1.838 – 128.063** | **0.012** |  |
| Degenerative MR | 2.758 | 1.145 – 6.643 | 0.024 | 1.675 | 0.603 – 4.653 | 0.322 |  |

*M-TEER: mitral valve transcatheter edge-to-edge repair; CI: confidence interval; MR: mitral regurgitation*

Supplemental Table 4: Logistic regression for prediction of absence of right-ventricular reverse remodeling within 3 months after M-TEER including NT-proBNP

|  | Univariate Logistic Regression | | | Multivariate Logistic Regression | | | |
| --- | --- | --- | --- | --- | --- | --- | --- |
|  | Odds Ratio | 95% CI | p | Odds Ratio | 95% CI | p |  |
| IGFBP-2 | 1.918 | 1.083 – 3.397 | 0.026 | excluded due to correlation with NT-proBNP | | |  |
| Procedural Success | 0.374 | 0.091 – 1.540 | 0.173 | 0.534 | 0.101 – 2.808 | 0.458 |  |
| NT-proBNP  (per 1,000 pg/ml) | 1.042 | 0.981 – 1.106 | 0.179 | 1.041 | 0.980 – 1.106 | 0.187 |  |
| **Pulmonary Disease** | **17.694** | **2.213 – 141.487** | **0.007** | **11.424** | **1.372 – 95.120** | **0.024** |  |
| Degenerative MR | 2.758 | 1.145 – 6.643 | 0.024 | 1.503 | 0.533 – 4.240 | 0.441 |  |

*M-TEER: mitral valve transcatheter edge-to-edge repair; CI: confidence interval; MR: mitral regurgitation*
